# Divergent pathways of mango fractions in promoting metabolic health: from gut microbiota remodeling to direct systemic regulation

**DOI:** 10.3389/fnut.2026.1744331

**Published:** 2026-03-23

**Authors:** Shiyuan Cai, Ruijing Zhang, Ke Li, Xin Li, Xianhao Zhou, Yanjun Liu, Kun Wang, Hongli Liao, Chunli Su

**Affiliations:** 1 Department of Pharmacy, The People's Hospital of Miyi County, Panzhihua, China; 2 School of Pharmacy, Chengdu Medical College, Chengdu, China; 3 Key Laboratory of Structure-Specific Small Molecule Drugs at Chengdu Medical College of Sichuan Province, School of Pharmacy, Chengdu Medical College, Chengdu, China; 4 School of Public Health, Chengdu Medical College, Chengdu, China

**Keywords:** by-product valorization, functional food, gut microbiota, mango kernel, mango peel, mango pulp, metabolomics

## Abstract

To dissect the distinct metabolic regulatory mechanisms of mango pulp (MPP), peel (MPL), and kernel (MKE), this study integrated 16S rRNA sequencing and fecal metabolomics in a mouse model. We uncovered a clear bifurcation in their actions. MPP and MPL acted primarily by remodeling the gut microbiota. Specifically, MPP treatment enriched Bilophila, while MPL treatment enriched Staphylococcus. Both components altered fecal peptide and lipid metabolism. In contrast, MKE displayed a direct systemic action largely independent of colonic microbial fermentation independent of the microbiota. Despite minimal impact on the fecal metabolome, its bioactive saponins were linked to the modulation of host steroid hormone biosynthesis, suggesting direct absorption. These findings establish a dual-mechanism model for mango’s bioactivity, providing a scientific framework for developing its components—specifically the MPP and MPL as microbiota-modulating agents and the MKE as a direct systemic regulator.

## 1 Introduction

Mango (*Mangifera indica L.*) is a major tropical fruit produced and processed in large volumes worldwide. Mango processing generates substantial by-products, primarily peel (MPL) and kernel (MKE), which constitute 35–60% of the total fruit mass (1, 2). These materials are typically discarded, causing both resource waste and environmental concerns. Consequently, the valorization of mango by-products has become a research priority in food science and agricultural economics.

While the mango pulp (MPP) is prized for its flavor and nutritional value, being rich in sugars, vitamins, and carotenoids, the by-products are increasingly recognized as more concentrated sources of specific bioactive compounds (3–6). For instance, MPL contains exceptionally high levels of dietary fiber and phenolic compounds (notably mangiferin). The MKE is a valuable source of antioxidant polyphenols, such as gallic and ellagic acids, and unique lipids (2, 4, 6). These components have demonstrated potential health benefits, including antioxidant, anti-inflammatory, and antimicrobial properties.

The gut microbiota is now recognized as a critical mediator between diet and host health (7, 8). Dietary components like fiber and polyphenols, while largely indigestible by the host, serve as key substrates for gut microbes. Microbial fermentation of these compounds alters the composition and function of the gut microbiota. These microbial shifts, and the resulting changes in their metabolites (e.g., short-chain fatty acids, secondary bile acids), can regulate host energy metabolism, immune function, and the nervous system, thereby profoundly impacting overall health (9–11).

However, while the health benefits of fruits are generally accepted and the chemical profiles of mango parts are known, a key knowledge gap remains: how the commonly consumed MPP and the discarded by-products differentially influence host physiology by modulating the gut microbiota. Previous studies have been fragmented, often focusing on a single component (e.g., MPL). Crucially, a systematic, parallel comparison of MPP, MPL, and MKE under identical experimental conditions has been lacking, leaving it unclear whether they act through common or distinct mechanisms.

This study aimed to address this gap by systematically comparing the effects of MPP, MPL, and MKE. We hypothesized that these three components would uniquely modulate the host’s gut ecosystem and elicit distinct physiological responses. To test this, we used a mouse model and a multi-omics approach, integrating physiological analysis, 16S rRNA gene sequencing, and untargeted metabolomics. The specific objectives were to: (1) evaluate the distinct effects of MPP, MPL, and MKE on host physiological indicators; (2) characterize how each component alters the gut microbiota structure; and (3) connect these microbial changes to shifts in the host’s fecal metabolic profile, thereby establishing the mango component-gut microbiota-host metabolism axis and exploring the underlying mechanisms of their health benefits.

## 2 Materials and methods

### 2.1 Preparation of mango fractions (MPP, MPL, and MKE)

Fresh Guifei mangoes were obtained from Lvyang Guohang Co., Ltd. (Panzhihua, China) and ripened at room temperature (23 ± 2 °C) until soft to the touch. The ripened mangoes were then manually separated into pulp, peel, and kernel. For the MPP and MPL, the materials were processed separately. Both were frozen at −80 °C for 12 h and then lyophilized for 48 h. The resulting dried materials were pulverized and sieved to a uniform particle size of 500–600 μm. The final powders were stored in a desiccator until use. For the MKE, the fresh MKE were dried in an oven at 40 °C for 7 days and subsequently pulverized into a powder. This powder was then extracted with 70% ethanol (1:20, w/v) using a Soxhlet apparatus. The extraction was performed at 30 °C for 2 h per cycle and repeated until the refluxing solvent was colorless. The combined extracts were concentrated by rotary evaporation at 55 °C, lyophilized to yield the final powder (MKE), and stored at −80 °C until use.

### 2.2 Animal experiment and sample collection

Forty-eight male Kunming mice (40 ± 2 g) were obtained from the Experimental Animal Center of Chengdu Medical College. The mice were housed under standard conditions (23 ± 2 °C, 60 ± 10% humidity, 12 h light/dark cycle) with ad libitum access to chow and water. All animal procedures received approval from the Ethics Committee of Chengdu Medical College (Approval no. 2024-N0153).

Following a 7-day acclimatization, the mice were randomly divided into four groups (*n =* 12 per group) for a 30-day intervention. The groups received a daily gavage as follows: the control (Ctrl) group received 0.3 mL of distilled water; the MPP group, 12.48 mg of MPP powder; the MPL group, 21.12 mg of MPL powder; and the MKE group, 4.40 mg of MKE.

All suspensions were freshly prepared in 0.3 mL of purified water immediately before administration. The dosages for each group were meticulously calculated to simulate a realistic dietary intake scenario for mice. This approach ensures that the administered dose of each fraction (MPP, MPL, and MKE) corresponds to an equivalent amount of the original fresh material, effectively normalizing the intervention by the natural yield of each mango part. The calculation was based on the following assumptions and steps: (1) The average daily food intake of a mouse was estimated as 3% of its body weight. Given the average mouse weight of 44.64 g, the daily food intake was approximately 1.34 g. (2) The proportion of fruit in the diet was assumed to be 8% of total food intake. (3) The yields from fresh mango to dry material were factored in. The specific calculations were as follows. MPP group (pulp dry matter yield: 11.65%): D_MPP_ = (44.64 g × 3% × 8%) × 11.65% × 1,000 mg/g ≈ 12.48 mg. MPL group (pulp dry matter yield: 19.71%): D_MPL_ = (44.64 g × 3% × 8%) × 19.71% × 1,000 mg/g ≈ 21.12 mg. MKE group (kernel dry weight: 33.21%; extract yield: 12.31%): As mango kernel is not typically consumed directly, an ethanol extract was used. The dose was calculated by further factoring in the kernel’s dry weight and the final extract yield. D_MKE_ = (44.64 g × 3% × 8%) × 33.21% × 12.31% × 1,000 mg/g ≈ 4.40 mg. Throughout the intervention, body weight and general health were monitored. At the end of the experiment, fresh fecal samples were collected, snap-frozen in liquid nitrogen, and stored at −80 °C for subsequent multi-omics analysis. For the multi-omics analyses, a representative subset of samples was selected from the full cohort (*n =* 12 per group) for reasons of cost-effectiveness. To ensure the selected samples were representative of the group’s overall physiological response, we selected mice whose final weight gain percentages were closest to the mean weight gain of their respective group. From each group, six such representative mice were selected for fecal metabolomics analysis (*n =* 6). From this subset of six, four mice per group were then used for 16S rRNA gene sequencing (*n =* 4) (12, 13).

### 2.3 16S rRNA gene sequencing and bioinformatics

Total microbial DNA was extracted from fecal samples using a commercial kit. The V3-V4 hypervariable region of the 16S rRNA gene was amplified with universal primers. The resulting PCR products were purified, quantified, and pooled to construct a sequencing library, which was sequenced on an Illumina MiSeq platform (Illumina, CA, USA) at Majorbio Bio-pharm Technology Co., Ltd. (Shanghai, China).

Raw sequencing data were processed using the QIIME2 pipeline (v2020.6.0). After quality filtering and trimming, sequences were denoised into Amplicon Sequence Variants (ASVs) via DADA2. Taxonomy was then assigned to the ASVs against the Silva database (v138.1). Functional potential of the gut microbiota was predicted using PICRUSt2, which inferred the abundance of KEGG Orthologs (KOs) by mapping ASVs to the KEGG database (v20230830) to estimate metabolic pathway prevalence.

### 2.4 Sample preparation for metabolomics

Fecal samples (50 mg) were homogenized in 400 μL of a pre-chilled methanol:water solution (4:1, v/v) containing L-2-chlorophenylalanine as an internal standard. After ultrasonication and centrifugation (13,000 g, 15 min, 4 °C), the supernatants were collected. A quality control (QC) sample was prepared by pooling equal aliquots from all supernatants.

### 2.5 UHPLC–MS/MS analysis

Metabolite separation was achieved on an ACQUITY UPLC HSS T3 column (100 mm × 2.1 mm, 1.8 μm; Waters, Milford, USA). The mobile phase consisted of solvent A (95% water + 5% acetonitrile with 0.1% formic acid) and solvent B (47.5% acetonitrile + 47.5% isopropanol + 5% water with 0.1% formic acid). The flow rate was 0.40 mL/min, the column temperature was set to 45 °C, and the injection volume was 10 μL. The specific elution gradient will be provided in the Supplementary materials.

The mass spectrometer was operated in both positive and negative ion modes using an electrospray ionization (ESI) source. The mass scan range was set from m/z 50 to 1,200. Key ESI source parameters were as follows: Ion Source Gas 1 (GS1) at 50 psi, Ion Source Gas 2 (GS2) at 50 psi, Curtain Gas (CUR) at 35 psi, and source temperature at 500 °C. The IonSpray Voltage Floating (ISVF) was set to +5,500 V for positive mode and −4,500 V for negative mode. The declustering potential (DP) was 80 V, and the collision energy (CE) was 40 ± 20 eV. The interface heater was on. Metabolites were identified by matching their exact mass-to-charge ratio (m/z) and MS/MS fragmentation patterns against public databases (e.g., KEGG, HMDB).

### 2.6 Statistical and multi-omics integrated analysis

All statistical analyses were performed in R (v4.1.2) or on the Majorbio Cloud Platform,^1^ with statistical significance set at *p <* 0.05.

For microbiota data, alpha diversity indices (ACE, Chao, Shannon, Simpson) were compared among groups using the Kruskal-Wallis H test for overall comparison, followed by pairwise Wilcoxon rank-sum test between treatment and control groups. Beta diversity was assessed by Principal Coordinate Analysis (PCoA) based on Bray–Curtis dissimilarity, weighted UniFrac, and unweighted UniFrac distances, with group significance tested by Permutational Multivariate Analysis of Variance (PERMANOVA). Differentially abundant bacterial taxa were identified using Linear Discriminant Analysis Effect Size (LEfSe). A Random Forest model identified the most important ASVs for discriminating among groups, and Circos diagrams visualized the distribution of dominant taxa.

For metabolomics data, unsupervised Principal Component Analysis (PCA) visualized overall metabolic differences. Differential metabolites were identified using a multi-step procedure. For each treatment group versus the control group, Orthogonal Partial Least Squares-Discriminant Analysis (OPLS-DA) was first applied. Metabolites with a Variable Importance in Projection (VIP) score > 1.0 from the OPLS-DA model were selected as potential candidates. The statistical significance of these candidates was then evaluated: after assessing for normality using the Shapiro–Wilk test, a Student’s *t*-test was used for normally distributed data, while a non-parametric Wilcoxon rank-sum test was applied for non-normally distributed data. The resulting *p*-values were then adjusted for multiple comparisons using the Benjamini-Hochberg method to control the False Discovery Rate (FDR). A metabolite was considered significantly different if it had a VIP > 1.0 and an FDR-adjusted *p* < 0.05. Venn diagrams illustrated the overlap of these metabolites among groups, and pathway enrichment analysis was conducted against the KEGG database using Fisher’s exact test.

For integrated multi-omics analysis, a Procrustes analysis first assessed the correlation between microbiota composition (ASV level) and the fecal metabolic profile. Spearman’s rank correlation was then used to identify specific associations between the relative abundances of key differential genera and the normalized intensities of differential metabolites. These associations were visualized as a correlation heatmap.

## 3 Results

### 3.1 Mango components differentially affect host physiological state

**Figure 1:**
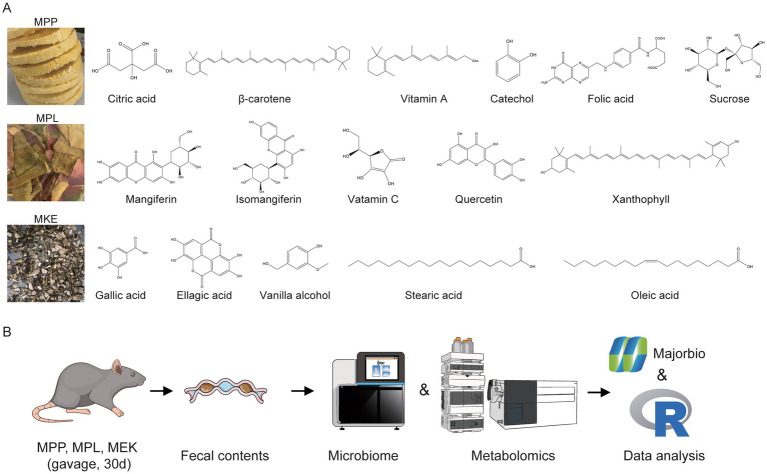
Distinct chemical profiles of mango components and the experimental design of the study. **(A)** Schematic representation of the primary chemical constituents identified in MPP, mango MPL, and MKE. MPP is characterized by sugars and organic acids, while MPL and MKE are rich in specific polyphenols like mangiferin and gallic acid, respectively. **(B)** Overview of the experimental workflow, illustrating the 30-day gavage intervention in mice with MPP, MPL, or MKE, followed by the collection of fecal samples for integrated 16S rRNA microbiome and untargeted metabolomics analyses.

The chemical profiles of MPP, MPL, and MKE are distinct (Figure 1A; Table 1) (14, 15). MPP consists mainly of sugars, whereas the by-products, MPL and MKE, are concentrated sources of functional compounds. MPL is particularly rich in dietary fiber and mangiferin, while MKE contains high levels of gallic and ellagic acids (14–17).

**Table 1:** Major components of MPP, MPL, and MKE.

Compound (Per 100 g)	MPP	MPL	MKE
Water (g)	83.46	72.5	9.1
Energy (kcal)	60	–	327
Carbohydrate, by difference (g)	14.98	28.2	18.2
Protein (g)	0.82	3.6	6.61
Total lipid(fat) (g)	0.38	2.2	9.4
Sugers, total (g)	13.99	25	70
Total Dietary fiber (g)	1.6	40–72.5	2.8
Minerals (mg)			
Calcium (Ca)	11	150	450
Iron (Fe)	0.16	40.6	11.9
Magnesium (Mg)	10	100	100
Phosphrous (P)	14		140
Potassium (K)	168	75	365
Sodium (Na)	1	50	150
Zinc (Zn)	0.09	1.74	1.1
Copper (Cu)	0.04–0.32	10.4	–
Selenium (Se)	0–0.6	–	–
Vitamins			
Vitamin C(total ascorbic acid, mg)	36.4	18–257	17
Thiamin (mg)	0.028	–	0.08
Riboflavin (mg)	0.038	–	0.13
Niacin (mg)	0.669	–	0.19
Pantothenic acid (mg)	0.119	–	0.12
Folate, dietary folate equivalents (μg)	43	–	–
Vitamin A, retinol activity equivalents (μg)	54	100	–
Vitamin E (α-tocopherol, mg)	0.9	0.25–0.59	1.3
Vitamin A (IU)	1,082	–	15
Vitamin K (phylloquinone, μg)	4.2	–	59
Vitamin B12	–	–	0.12
Organic acids			
Citric acid (%)	0.7	–	–
Mallic acid (%)	0.5	–	–
Polyphenols			
Cyanidin (mg)	0.1	–	–
Catechin (mg)	1.7	–	–
Kaempferol (mg)	0.1	3.6	-
Myricetin (mg)	0.1	–	–
Proanthocyanidin dimers (mg)	1.8	–	–
Peoanthocyanidin trimers (mg)	1.4	–	–
Gallic acid	0.69	–	23–838
Ellagic acid	–	–	3–156
Coumarin	–	–	12.7
Vanillin	–	–	202
Cinnamic acid	–	–	11.2
Ferulic acid	–	–	10.4
Mangiferin (mg)	–	169	4.2
Mangiferin gallate	–	321	–
Isomangiferin	–	13.4	–
Isomangiferin gallate	–	82	–
Quercetin	2.2	6.5	–
Tannins	–	–	20.7
Anthocyanins	–	360–565	–
Cyaniding	–	22.1	–
Pelargonidins	–	22.73	–
Delphinidins	–	18.02	–
Petunidins	–	21.6	–
Peonidins	–	24.42	–
Cartenoids (μg)			
β-carotene	640	1,310	–
α-carotene	9	–	–
β-cryptoxanthin	10	600	–
Lutein and zeaxanthin	23	299	–

Based on these compositional differences, we investigated the effects of each component on weight gain in mice over a 30-day study (Figure 1B). The Ctrl group exhibited a 15.68% gain in body weight (Table 2). In contrast, all mango-supplemented groups showed attenuated weight gain. The effect was modest in the MPP group, which gained 11.19%. However, the inhibition was significantly more pronounced in the by-product groups, with weight gain limited to 7.48% for the MPL group and 6.92% for the MKE group.

**Table 2:** Changes in body weight of mice.

Groups	Original weight (g)	The final weight (g)	Percentage of weight gain (%)
Ctrl	44.6 ± 0.35	51.6 ± 1.81	15.69 ± 2.51%
MPP	43.9 ± 0.32	48.8 ± 1.87	11.16 ± 2.07%
MPL	46.4 ± 0.26	49.9 ± 0.49	7.54 ± 0.57%
MKE	47.0 ± 0.15	50.2 ± 2.44	6.81 ± 3.07%

These data show that MPP, MPL, and MKE differentially regulate host weight. The stronger inhibitory effects of the by-products (MPL and MKE) suggest that their high content of dietary fiber and polyphenols may be is a key driver of this physiological outcome. This finding prompted our investigation into the underlying microbial mechanisms.

### 3.2 Mango components differentially modulate gut microbiota structure

**Figure 2:**
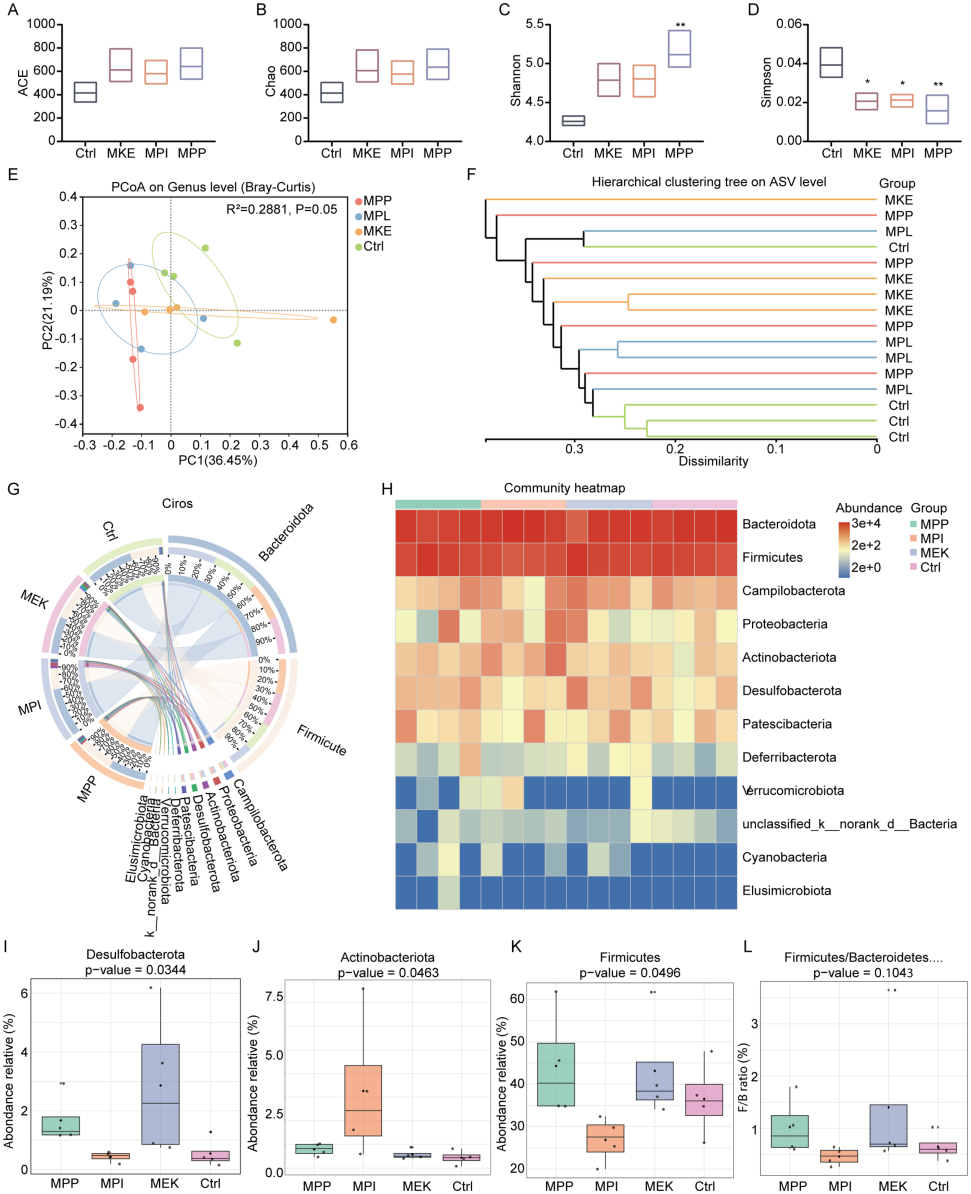
Mango components differentially modulate the gut microbiota structure and composition. **(A–D)** Alpha diversity analysis of the gut microbiota, showing the **(A)** ACE, **(B)** Chao, **(C)** Simpson, and **(D)** Shannon indices. All three mango-supplemented groups exhibited higher microbial richness and diversity compared to the Ctrl group. **(E)** Principal coordinate analysis (PCoA) based on Bray–Curtis dissimilarity at the genus level, illustrating a clear separation of the microbial community structures of the mango-treated groups from the Ctrl group (PERMANOVA, *p* = 0.05). **(F)** Hierarchical clustering tree at the ASV level, confirming the distinct clustering of intervention groups away from the Ctrl. **(G)** Stacked bar plot of the relative abundance of dominant bacterial phyla. **(H)** Community heatmap visualizing phylum-level abundance patterns across individual samples. **(I–L)** Statistical comparison of the relative abundances of key phyla that were significantly modulated by specific mango components: **(I)** Desulfobacterota, **(J)** Actinobacteriota, **(K)** Firmicutes, and **(L)** the Firmicutes-to-*Bacteroidetes* (F/B) ratio. Data are presented as mean ± SEM. For alpha diversity **(A–D)**, overall significance across groups was first assessed by the Kruskal-Wallis test. Pairwise comparisons against the Ctrl group were then performed using the Wilcoxon rank-sum test. *p < 0.05, **p < 0.01 vs. Ctrl group.

To determine how different mango components affect the gut microbiota, we performed 16S rRNA gene sequencing on fecal samples (Supplementary Table S1). First, we assessed *α*-diversity (Figures 2A–D; Supplementary Table S2). Compared to the Ctrl, all three mango-supplemented groups (MPP, MPL, and MKE) showed increased microbial richness and diversity. Specifically, the ACE and Chao richness indices were higher in all treatment groups (Figures 2A,B). Similarly, the Shannon diversity index was elevated, while the Simpson index was significantly lower (*p <* 0.05), indicating that mango supplementation enhanced community evenness and reduced the dominance of any single species (Figures 2C,D).

Next, *β*-diversity analysis was used to evaluate overall shifts in microbial community structure. To provide a comprehensive assessment, we performed Principal Coordinate Analysis (PCoA) using three distinct distance metrics. The analysis based on Bray–Curtis dissimilarity, which primarily reflects taxon abundance, showed a notable separation of the treatment groups from the Ctrl group (PERMANOVA, R^2^ = 0.288, *p* = 0.05), indicating a shift in the abundance of shared taxa (Figure 2E). To incorporate phylogenetic information, we also conducted PCoA using UniFrac distances (Supplementary Figure S1). The unweighted UniFrac analysis, which is sensitive to changes in rare taxa, revealed a statistically significant separation between the groups (PERMANOVA, R^2^ = 0.293, *p* = 0.015). This suggests that the interventions successfully altered the presence or absence of specific, including low-abundance, phylogenetic lineages. In contrast, the weighted UniFrac analysis, which accounts for both phylogeny and abundance, did not show a significant separation (PERMANOVA, R^2^ = 0.275, *p* = 0.097), implying that the core, most abundant phylogenetic branches of the community were more stable. This separation was corroborated by hierarchical clustering analysis, where Ctrl samples clustered together and formed a distinct branch from the mango-supplemented groups (Figure 2F). Together, these results demonstrate that mango intake drives community-wide shifts in the gut microbiota.

We then examined compositional changes at the phylum level. All groups were dominated by Firmicutes and Bacteroidota (Figure 2G). However, a community heatmap revealed substantial differences in their relative abundances across the groups (Figure 2H). Overall, all mango-treated groups displayed a trend toward a lower abundance of Firmicutes and a higher abundance of Bacteroidota compared to the Ctrl.

Statistical analysis of key phyla highlighted the specific effects of each mango component (Figures 2I–L). Only the MPL group significantly reduced the relative abundance of Firmicutes (*p =* 0.0496) and, consequently, exhibited the lowest Firmicutes-to-Bacteroidota (F/B) ratio (Figures 2K,L). The MPL group also uniquely and significantly increased the abundance of the beneficial phylum Actinobacteriota (*p =* 0.0463) (Figure 2J). In contrast, the MKE group exerted a different regulatory effect, significantly increasing the abundance of Desulfobacterota (*p =* 0.0344) (Figure 2I).

In summary, mango supplementation reshapes the gut microbial ecosystem by increasing diversity, altering the overall community structure, and modulating the abundance of key phyla in a component-specific manner.

### 3.3 Mango components induce specific shifts in key bacterial taxa

**Figure 3:**
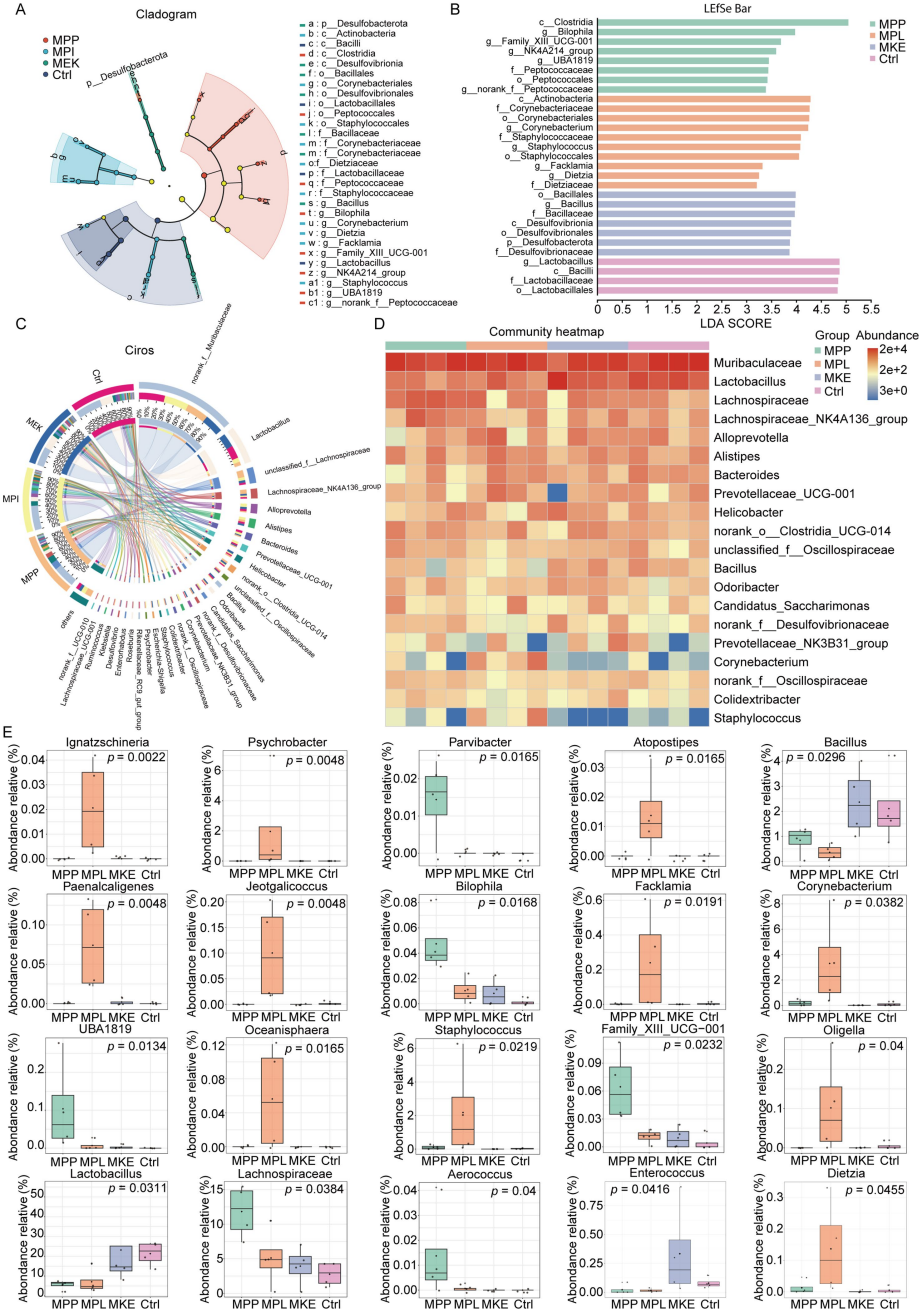
Mango components induce specific and distinct shifts in key bacterial taxa. **(A, B)** Linear discriminant analysis effect size (LEfSe) analysis identifying the key bacterial taxa that serve as biomarkers for each group. **(A)** The cladogram illustrates the phylogenetic distribution of these biomarker taxa. **(B)** The bar plot displays the linear discriminant analysis (LDA) scores, indicating the effect size of the differentially abundant taxa. Taxa are colored according to the group in which they are most abundant. **(C)** Stacked bar plot showing the relative abundance of the most prevalent genera across the different groups. **(D)** Heatmap displaying the relative abundance of key genera in each individual sample, highlighting distinct microbial signatures, such as the marked suppression of the family Muribaculaceae in the MKE group. **(E)** Statistical comparisons of the relative abundances of representative differential genera. This highlights the selective enrichment of *Bilophila* and Lachnospiraceae by MPP, the significant bloom of rare genera like *Staphylococcus*, *Psychrobacter*, and *Corynebacterium* by MPL, and the suppression of *Lactobacillus* by both MPP and MPL. Data are presented as mean ± SEM. Statistical significance was determined by the Wilcoxon rank-sum test.

To identify the specific microbial taxa driving the observed community shifts, a Linear Discriminant Analysis Effect Size (LEfSe) analysis was conducted. The results revealed that each mango component cultivated a unique microbial fingerprint (Figures 3A,B; Supplementary Table S3). Notably, the primary biomarker for the Ctrl group was the entire *Lactobacillus* lineage, indicating that its abundance was highest in the untreated state and was suppressed by all mango interventions. For the treatment groups, MPL was characterized by the significant enrichment of two distinct lineages: the class Actinobacteria (specifically the order Corynebacteriales, including *Corynebacterium* and *Dietzia*) and the genus *Staphylococcus*. MPP selectively promoted taxa within the class Clostridia, with *Bilophila* showing the highest LDA score. The MKE was primarily associated with biomarkers from the order Bacillales (specifically *Bacillus*) and the phylum Desulfobacterota.

Analysis of dominant taxa provided further insights into these regulatory patterns (Figures 3C,D). The family Muribaculaceae was identified as the most dominant taxon in the Ctrl, MPP, and MPL groups. However, its abundance was significantly suppressed by the MKE intervention. Conversely, the abundance of *Lactobacillus*, the second most dominant genus in the Ctrl group, was sharply reduced by both MPP and MPL treatment but was maintained at a level comparable to the Ctrl in the MKE group. Furthermore, the MPP intervention was unique in its significant promotion of the Lachnospiraceae family.

Statistical analysis of differential genera quantified these specific regulatory effects (Figure 3E). The MPL intervention triggered the most dramatic change, inducing a significant bloom of numerous low-abundance or rare genera, including *Ignatzschineria* (*p =* 0.0022), *Psychrobacter* (*p =* 0.0048), *Staphylococcus* (*p =* 0.0219), and *Corynebacterium* (*p =* 0.0382). In contrast, MPP supplementation selectively promoted an entirely different set of *bacteria*, most notably *Bilophila* (*p =* 0.0168) and members of the Lachnospiraceae family (*p =* 0.0384). A key finding common to both MPP and MPL interventions was the significant suppression of both *Lactobacillus* (*p =* 0.0311) and *Bacillus* (*p =* 0.0296) compared to the Ctrl group.

In summary, the MPP, MPL, and MKE of the mango exert highly distinct regulatory effects on the gut microbiota. The MPL acts as a potent remodeler by inducing a bloom of rare taxa. The MPP selectively enriches for another class of *bacteria*, including Lachnospiraceae. The MKE’s primary influence is the suppression of the dominant Muribaculaceae family. Their common effect involves the suppression of *Lactobacillus*, revealing complex and component-specific pathways for reshaping the gut microbial ecosystem.

### 3.4 Key bacterial signatures drive functional shifts in carbohydrate metabolism

**Figure 4:**
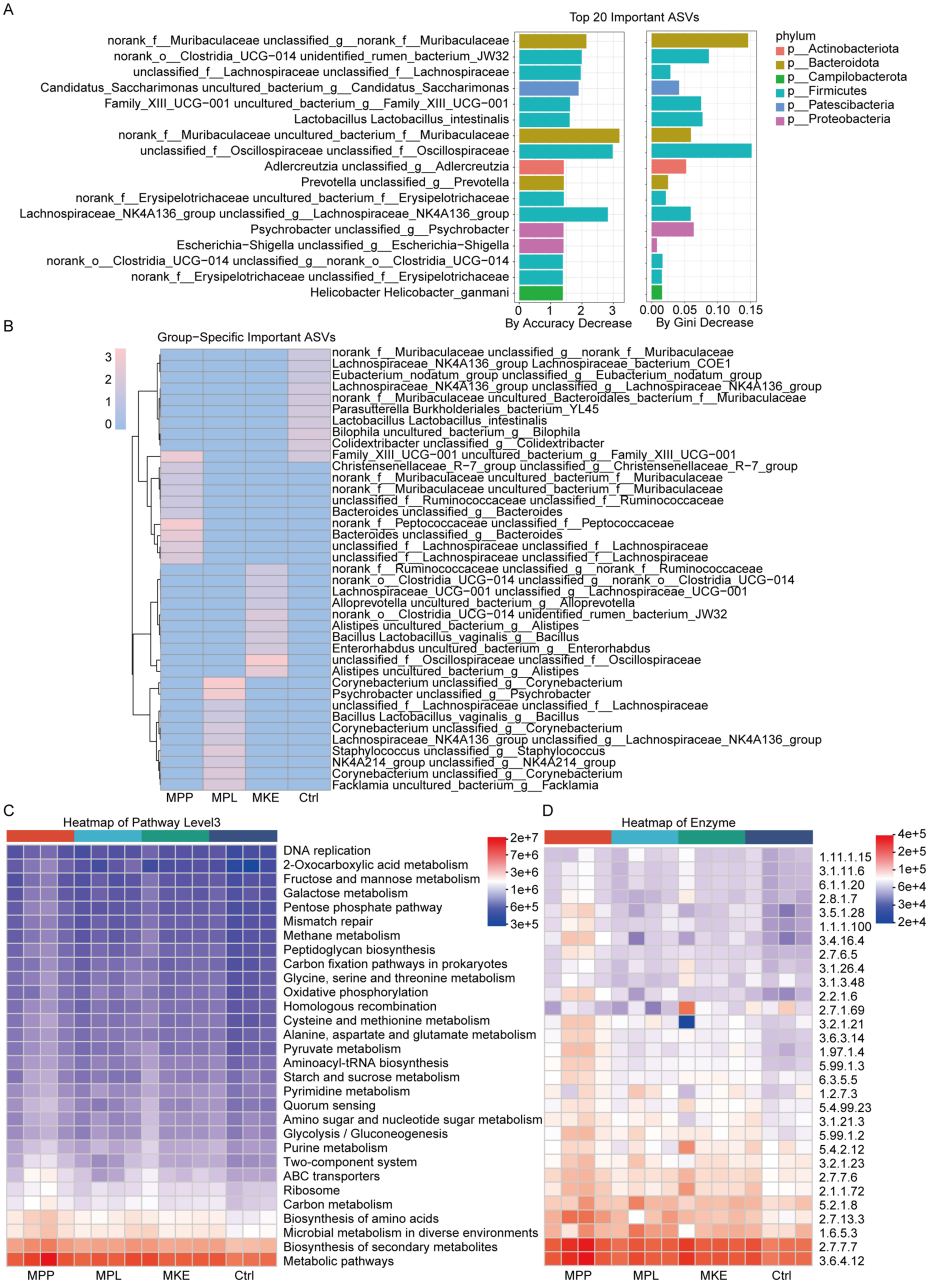
Key bacterial signatures drive functional shifts in microbial carbohydrate metabolism. **(A)** Random forest analysis identifying the top 20 most important amplicon sequence variants (ASVs) for discriminating between the experimental groups, ranked by mean decrease in Gini impurity. Members of the families *Muribaculaceae*, *Oscillospiraceae*, and *Lachnospiraceae* were among the most influential biomarkers. **(B)** Heatmap displaying the relative abundance of these top 20 discriminatory ASVs across individual samples. This visualization confirms the component-specific regulatory effects, such as the enrichment of ASVs from the *Lachnospiraceae NK4A136 group* in the MPP group and a distinct cluster of rare taxa (e.g., *Psychrobacter*, *Corynebacterium*) in the MPL group. **(C)** Heatmap of predicted microbial functions (KEGG Level 3 pathways), inferred using PICRUSt2. The analysis shows that microbial functions in the MPP and MPL groups were significantly altered, particularly with the upregulation of pathways related to carbohydrate metabolism (e.g., fructose and mannose metabolism, galactose metabolism). **(D)** Heatmap of the predicted abundance of key enzymes (EC numbers), revealing the molecular basis for the functional shifts. The MPP group showed a higher abundance of genes encoding glycoside hydrolases, such as *β*-galactosidase (EC 3.2.1.23) and β-glucosidase (EC 3.2.1.21), and DNA replication enzymes (e.g., DNA polymerase, EC 2.7.7.7).

To pinpoint the most influential taxa responsible for differentiating the treatment groups, we applied a Random Forest machine learning model. The model identified the top 20 amplicon sequence variants (ASVs) that were most critical for distinguishing the groups, with ASVs from the family Muribaculaceae emerging as by far the most critical biomarkers, exhibiting the highest decrease in model accuracy upon removal. Other highly influential taxa included members of the families *Oscillospiraceae* and Lachnospiraceae (Figure 4A). This data-driven approach confirmed that a small subset of key *bacteria* is sufficient to explain the majority of the community-wide shifts.

A heatmap of these key ASVs visually confirmed the highly specific regulatory effects of each mango component (Figure 4B). This analysis revealed a distinct “bacterial fingerprint” for each group. The MPL intervention induced a pronounced bloom of a specific cluster of rare taxa (including *Psychrobacter*, *Corynebacterium*, and *Staphylococcus*), which were nearly absent in all other groups. Conversely, MPP specifically promoted a different set of ASVs, primarily from the Lachnospiraceae *NK4A136 group* and *Bilophila*. Furthermore, the model reiterated a common suppressive effect of both MPP and MPL on *Lactobacillus intestinalis* and *Bacillus*.

Next, to understand the functional consequences of these microbial shifts, we used PICRUSt2 to predict the metabolic potential of the communities. The complete list of all predicted KEGG Orthologs (KOs) and the full statistical results of the differential abundance analysis are provided in Supplementary Tables S4, S5. A KEGG pathway analysis revealed that while many core housekeeping pathways were upregulated across all mango treatment groups, the microbial functions in the MPP and MPL groups were most significantly altered compared to the Ctrl (Figure 4C). The most prominently upregulated pathways in both MPP and MPL groups were related to carbohydrate metabolism, particularly fructose and mannose metabolism and galactose metabolism. This strongly suggests that the microbiota adapted its functional capacity to utilize the specific carbohydrates abundant in mango.

To uncover the molecular basis for this functional shift, we analyzed the abundance of genes encoding key enzymes (Figure 4D). This revealed a coordinated upregulation of enzymes in several key functional classes, particularly in the MPP and MPL groups. Crucially, this included a higher abundance of glycoside hydrolases such as *β*-glucosidase (EC 3.2.1.21) and β-galactosidase (EC 3.2.1.23), the “tools” required to break down complex plant-derived polysaccharides. Concurrently, an increased abundance of genes for DNA/RNA replication and nucleotide synthesis (e.g., DNA/RNA polymerases, DNA topoisomerase) and ABC transporters (for nutrient uptake) was observed. This is consistent with the active proliferation and metabolic activity of the specific bacteria enriched by mango MPP and MPL.

In summary, these results link species-level shifts to functional consequences. Mango intake, particularly of MPP and MPL, reshapes the gut microbiota’s metabolic profile by selectively modulating key bacterial taxa. The core mechanism involves enriching for bacteria equipped with a robust enzymatic toolkit for carbohydrate decomposition, thereby enhancing the community’s overall capacity to metabolize the complex plant-derived polysaccharides abundant in mango.

### 3.5 MPP and MPL, but not MKE, profoundly reshape the host fecal metabolome

**Figure 5:**
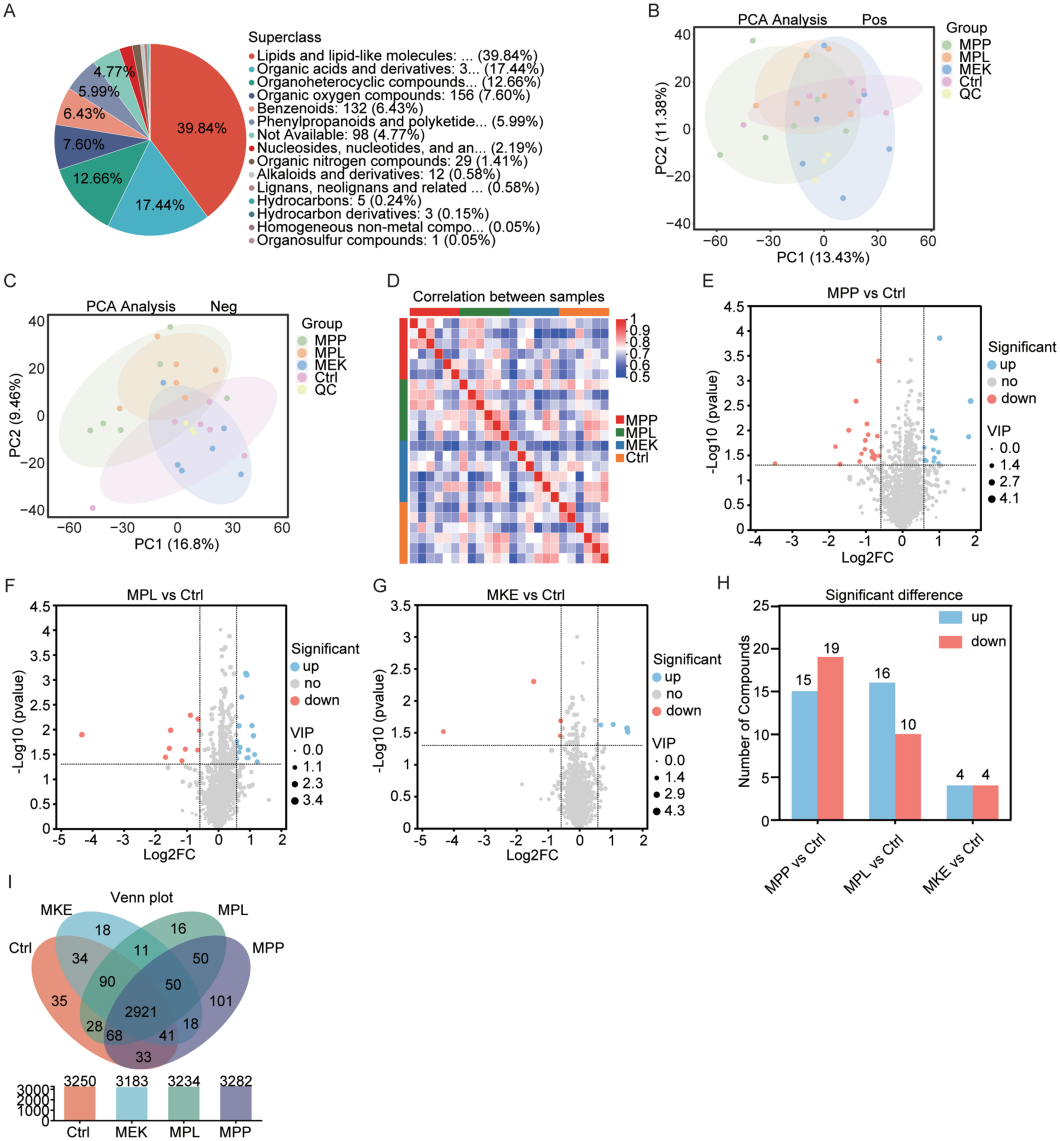
MPP and MPL, but not MKE, profoundly reshape the host fecal metabolome. **(A)** Pie chart illustrating the classification of all detected metabolites at the superclass level, with lipids and lipid-like molecules being the most abundant category. (B, C) Principal component analysis (PCA) plots of fecal metabolites in **(B)** negative and **(C)** positive ion modes. The analysis shows a clear separation of the metabolic profiles of the MPP and MPL groups from the Ctrl and MKE groups. **(D)** Heatmap of inter-sample correlations, demonstrating that the metabolic profiles of the MPP and MPL groups have lower similarity to the Ctrl, whereas the MKE group’s profile remains highly similar to the Ctrl. **(E–G)** Volcano plots showing the differentially abundant metabolites in the **(E)** MPP vs. Ctrl, **(F)** MPL vs. Ctrl, and **(G)** MKE vs. Ctrl comparisons. Red and blue dots indicate significantly upregulated and downregulated metabolites, respectively (VIP > 1.0, *p* < 0.05). **(H)** Bar chart quantifying the total number of significantly different metabolites, highlighting the substantial metabolic impact of MPP and MPL compared to the minimal changes induced by MKE. **(I)** Venn diagram illustrating the number of shared and unique metabolites detected across the four experimental groups.

To determine how mango-induced microbial shifts affected the host, we performed untargeted metabolomics on fecal samples. Lipids and lipid-like molecules were the most abundant chemical class detected, followed by organic acids and organoheterocyclic compounds (Figure 5A; Supplementary Tables S6, S7).

Multivariate analysis immediately revealed that MPP and MPL dramatically reshaped the host’s metabolic landscape. In Principal Component Analysis (PCA), both the MPP and MPL groups separated distinctly from the Ctrl and MKE groups (Figures 5B,C). This clear separation was corroborated by inter-sample correlation analysis, which showed that the metabolic profiles of the MPP and MPL groups had low similarity to the Ctrl, while the MKE group remained highly similar to it (Figure 5D).

Quantification of differentially abundant metabolites confirmed the magnitude of these changes. Compared to the Ctrl, the MPP group had 34 differential metabolites (15 upregulated, 19 downregulated) and the MPL group had 26 (16 upregulated, 10 downregulated). In stark contrast, the MKE group had only 8 (Figures 5E–H). The full list of all detected metabolites and the complete statistical results for the differential analysis are provided in Supplementary Table S8. A Venn diagram further showed that these changes were driven by shifts in the abundance of shared metabolites rather than the appearance of new ones (Figure 5I). For MPP and MPL, these metabolomic findings are consistent with the microbiome data, suggesting metabolic remodeling is a direct consequence of microbial shifts. However, the minimal changes in the MKE group present a clear paradox.

**Figure 6:**
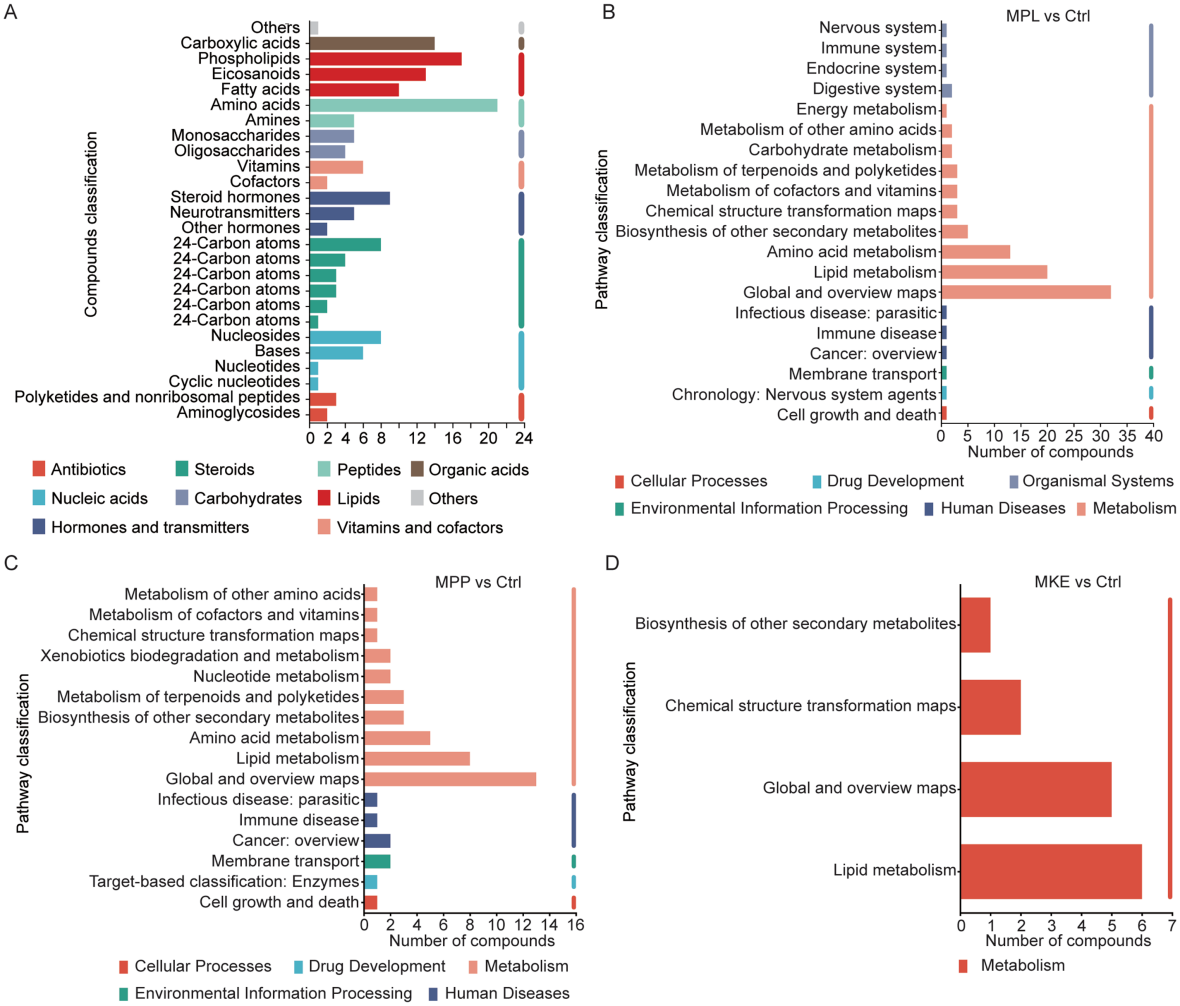
Functional pathway analysis reveals the distinct metabolic roles of MPP, MPL, and MKE. **(A)** Bar chart showing the chemical classification of all differential metabolites identified across the comparisons, indicating that the metabolic changes are concentrated in key classes such as lipids, organic acids, and amino acids. **(B–D)** KEGG pathway enrichment analysis of the differential metabolites for the **(B)** MPL vs. Ctrl, **(C)** MPP vs. Ctrl, and **(D)** MKE vs. Ctrl comparisons. The analysis highlights the distinct functional profiles of each component. MPL shows the most extensive regulatory effect, with significant enrichment in pathways related to lipid metabolism, amino acid metabolism, the immune system, and the endocrine system. MPP shows a similar but less broad profile, primarily affecting lipid and amino acid metabolism. In contrast, MKE demonstrates a highly specific effect, with enrichment almost exclusively in lipid metabolism pathways.

A key finding, however, was the apparent paradox presented by the MKE. Despite being the most effective component for inhibiting weight gain (Figure 1), MKE induced the most minimal changes in the fecal metabolome. This suggests that the MKE employs a fundamentally different mechanism of action from the MPP and MPL. Rather than extensively remodeling the gut environment, its bioactive compounds—such as the saponins identified in Figure 6—are likely absorbed directly into circulation to act on systemic metabolic pathways and distal organs. From this perspective, the stability of the fecal metabolome is not a sign of inaction, but rather compelling evidence for a “direct absorption, systemic action” hypothesis for MKE, a pathway that appears to operate without major reliance on colonic microbial fermentation.

### 3.6 Functional analysis reveals distinct metabolic roles for MPP, MPL, and MKE

To understand the biological meaning of the metabolic shifts induced by mango, we first classified the differential metabolites. The changes were concentrated in key chemical classes, including amino acids, lipids (notably phospholipids and eicosanoids), organic acids, and steroids, indicating that mango primarily influences the host’s core amino acid, lipid, and energy metabolism (Figure 6A).

To pinpoint the specific biological pathways involved, we performed KEGG pathway enrichment analysis. This revealed clear and distinct functional profiles for each mango component (Figures 6B–D).

First, the regulation of lipid metabolism was a shared feature across all three components. However, the mango MPL and MPP induced broad changes, whereas the MKE’s effect was highly focused, affecting fewer metabolites within this pathway.

Second, the regulation of amino acid metabolism clearly distinguished the fleshy parts of the fruit from the MKE. Pathways for amino acid metabolism were significantly enriched in both the MPP and MPL groups, but were completely unaffected in the MKE group. This reinforces the MKE’s highly specific mode of action.

Finally, the MPL demonstrated the most powerful and extensive regulatory capacity. Uniquely, its metabolic signature was also significantly enriched in pathways related to the immune and endocrine systems, suggesting a potent, systemic impact that goes beyond that of the MPP and MKE. Direct comparison confirmed the MPP’s effects were similar to, but less potent and broad than, the MPL’s.

In summary, this pathway-level view defines clear functional identities for each mango component. The MPL acts as the most potent, broad-spectrum metabolic regulator. The MPP is a moderate, broad-spectrum regulator. In contrast, the MKE functions as a mild but highly specific modulator, targeted almost exclusively to lipid metabolism.

### 3.7 Saponins and peptides as key molecular drivers of mango’s distinct metabolic effects

**Figure 7:**
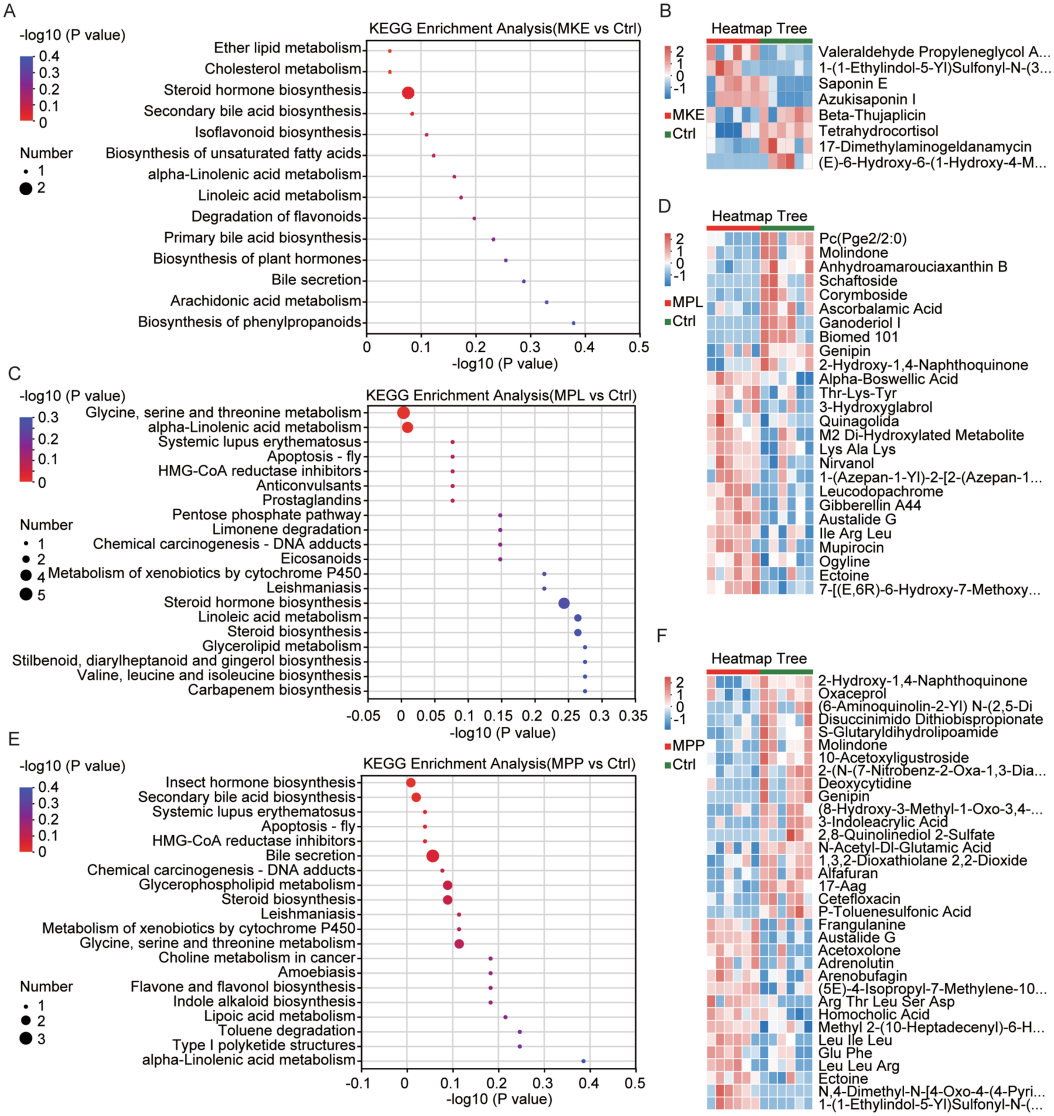
Molecular analysis identifies saponins and peptides as key drivers of the distinct metabolic effects of mango components. **(A,B)** Analysis of metabolites differentially regulated by MKE. **(A)** KEGG enrichment analysis reveals a highly targeted effect, with pathways almost exclusively related to steroid metabolism, including cholesterol metabolism and steroid hormone biosynthesis. **(B)** The heatmap provides a direct mechanistic link, showing the concurrent accumulation of MKE-derived saponins (e.g., saponin E, azukisaponin I) and the significant downregulation of the endogenous host metabolite tetrahydrocortisol. **(C,D)** Analysis of metabolites differentially regulated by MPL. **(C)** KEGG enrichment analysis demonstrates a potent and broad impact, with strong enrichment in lipid pathways like steroid biosynthesis. **(D)** The corresponding heatmap shows the significant upregulation of various peptides (e.g., Thr-Lys-Tyr), reflecting a profound impact on peptide metabolism. **(E, F)** Analysis of metabolites differentially regulated by MPP. **(E)** KEGG pathway enrichment analysis shows significant enrichment in pathways such as the metabolism of xenobiotics by cytochrome P450. **(F)** A heatmap of key differential metabolites reveals the upregulation of numerous peptides (e.g., Leu-Ile-Leu) and the downregulation of the microbially-derived anti-inflammatory molecule 3-indoleacrylic acid.

To identify the precise molecular drivers of these metabolic shifts, we examined the specific pathways and key metabolites altered by each mango component (Figure 7). This analysis revealed two fundamentally different modes of action.

First, the MKE demonstrated a highly targeted, systemic mechanism. Its metabolic impact was almost exclusively focused on steroid-related pathways, including cholesterol metabolism, bile acid biosynthesis, and steroid hormone biosynthesis (Figure 7A). The molecular data provided a clear mechanistic link for this effect (Figure 7B). We observed a significant accumulation of saponins (e.g., Saponin E, Azukisaponin I)—bioactive compounds originating from the MKE itself—in the host. Concurrently, Tetrahydrocortisol, a key downstream metabolite of host cortisol, was significantly downregulated. This provides a direct chain of evidence: bioactive saponins from the MKE appear to be absorbed and directly suppress the host’s steroid hormone synthesis pathway, linking an exogenous plant compound to a specific endogenous metabolic outcome.

In stark contrast, the MPP and MPL induced broad-spectrum changes consistent with a comprehensive remodeling of the host’s metabolic environment. A central feature for both was the profound disruption of amino acid and peptide metabolism, evidenced by the systematic upregulation of numerous di- and tripeptides (e.g., Leu-Ile-Leu in MPP, Thr-Lys-Tyr in MPL) (Figures 7D,F).

However, consistent with its more potent physiological effects, the MPL exerted a far more powerful and widespread effect than the MPP. Its metabolic signature showed an exceptionally strong enrichment in multiple lipid pathways, such as steroid biosynthesis and linoleic acid metabolism (Figure 7C). This potent modulation of host lipid networks, combined with its impact on peptide metabolism, distinguishes the peel as the most powerful modulator of the gut ecosystem among the tested components. The MPP’s effect, while significant, was more moderate. It notably enriched for the Metabolism of xenobiotics by cytochrome P450 pathway and uniquely led to a decrease in 3-Indoleacrylic Acid, an anti-inflammatory molecule derived from microbial tryptophan metabolism (Figures 7E,F).

In summary, the molecular data solidifies the distinct roles of each component. The MKE acts like a targeted drug, delivering specific bioactive compounds (saponins) to modulate a specific host pathway (steroid synthesis). The MPP and MPL act as broad-spectrum ecosystem modulators. The MPL is the most powerful agent, distinguished by its potent and widespread impact on host lipid metabolism. The MPP’s impact is uniquely characterized by a reduction in the microbially-derived anti-inflammatory molecule 3-Indoleacrylic Acid.

### 3.8 Correlation analysis confirms a dual-mechanism model for mango’s metabolic effects

**Figure 8:**
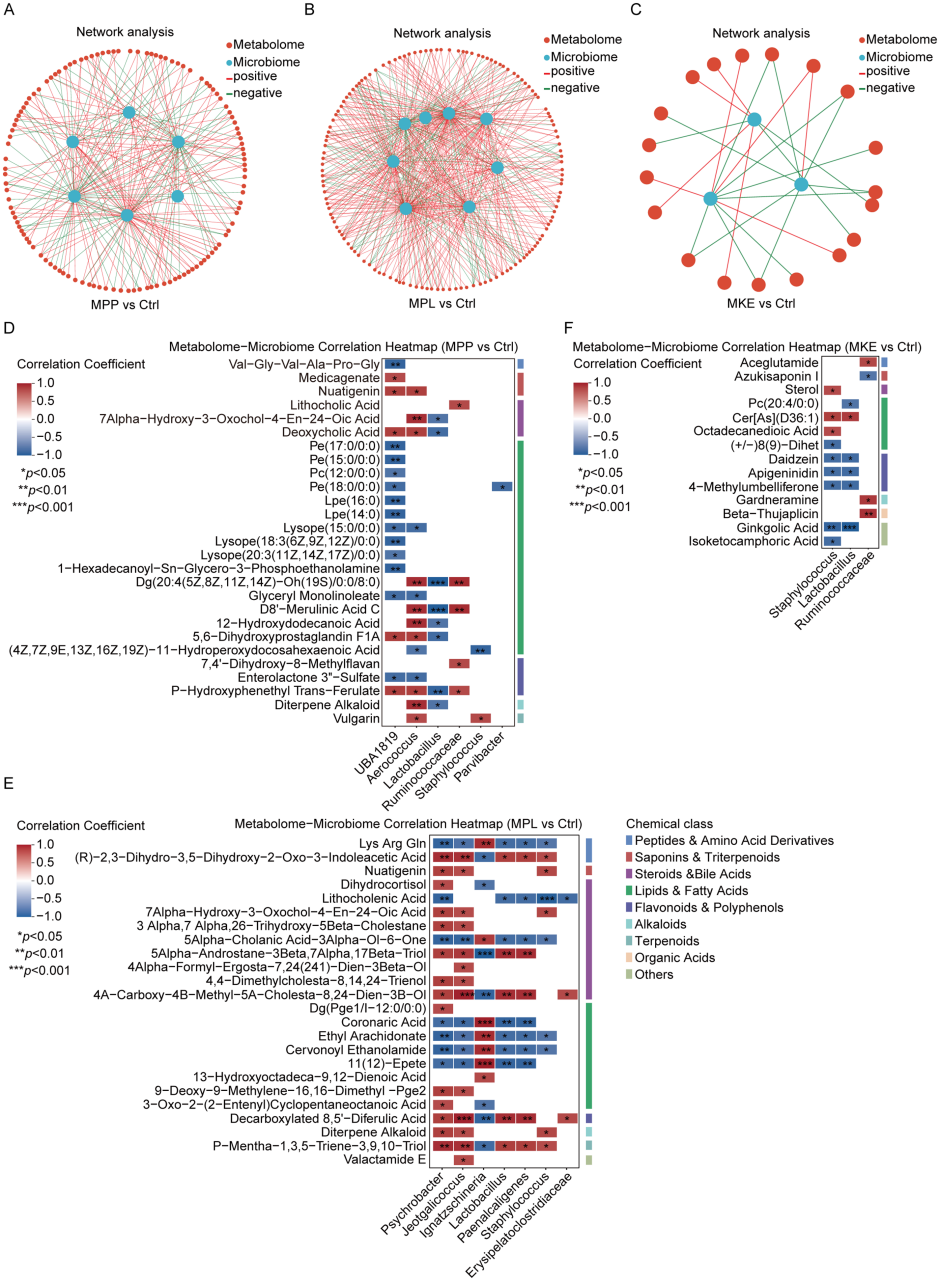
Correlation analysis links key bacterial shifts to host metabolic changes, confirming a dual-mechanism model. Spearman correlation heatmaps for the **(A)** MPP, **(B)** MPL, and **(C)** MKE groups, and corresponding network diagrams **(D–F)**. On the heatmaps, metabolites on the *y*-axis are grouped by chemical class (e.g., peptides, steroids and bile acids, lipids). Asterisks within the cells denote the level of statistical significance (*p < 0.05, **p < 0.01, ***p < 0.001), highlighting key microbe-metabolite associations. For MPP **(A,D)**, the enriched *Ruminococcaceae* family correlates positively with specific lipids. For MPL **(B,E)**, *Staphylococcus* correlates with peptides, while *Psychrobacter* correlates strongly with steroids and bile acids. For MKE **(C,F)**, the critical lack of significant correlations between key saponins and dominant bacteria supports a direct, systemic mechanism. In the networks, red lines indicate positive correlations and blue lines indicate negative correlations.

To establish the final mechanistic link between the microbial shifts (Figure 3) and the host metabolic signatures (Figure 6), we performed a correlation analysis (Figure 8). The results provide decisive evidence for a dual-mechanism model, distinguishing the microbially-mediated effects of the MPP and MPL from the direct, systemic action of the MKE.

First, the MPP and MPL were found to alter host metabolism by modulating the gut microbiota. The specific bacteria enriched by these components were significantly correlated with the observed metabolic changes.

For MPP: Analysis shows the enriched Ruminococcaceae family (the higher-level taxon for Lachnospiraceae, Figure 3) correlates strongly and positively with lipid-like molecules, including the diglyceride Dg (20:4.) and D8’-Merulinic Acid C (correlation coefficient = 0.943, *p* < 0.005) (Figures 8A,D). This strong correlation suggests that these bacteria may play a role in altering the gut’s lipid profile, which could be an upstream event contributing to broader metabolic shifts like the peptide upregulation seen in Figure 6.

For MPL: The data provides an even clearer link. The MPL-enriched Staphylococcus (a biomarker from Figure 3) is positively correlated with upregulated tripeptides (e.g., Lys Arg Gln). Furthermore, the bloom of Psychrobacter correlates strongly with a wide array of steroid and bile acid metabolites (Figures 8B,E). This provides a strong correlational link that helps to explain the MPL’s potent and widespread influence on host lipid and peptide metabolism (Figure 6).

In stark contrast, the analysis for the MKE substantiates the hypothesis of a direct effect on the host through a critical lack of microbe-metabolite associations.

We found no significant correlations between the MKE’s primary microbial impact (suppression of Muribaculaceae, Figure 3) and its primary metabolic outcome (suppression of host steroid pathways, Figure 6). Moreover, key bioactive compounds from the MKE found in the host, such as Azukisaponin I, showed no meaningful correlation with dominant fecal bacteria (Figures 8C,F). This absence of a statistical link is the crucial finding, as it lends strong support to the hypothesis that the MKE’s mechanism bypasses significant microbial mediation. It is consistent with the hypothesis that its bioactive saponins are absorbed to exert a direct, systemic effect on host steroid synthesis.

In summary, this analysis solidifies the dual-mechanism model. The MPP and MPL function by modulating the gut microbiota, enriching for specific bacteria (Ruminococcaceae, Staphylococcus, Psychrobacter) that in turn alter the host’s peptide and lipid metabolism. The MKE, conversely, acts through a direct, systemic mechanism where its bioactive saponins are absorbed by the host to affect metabolic pathways, with its impact on the microbiota being a secondary effect rather than the primary driver of its physiological benefits. The complete matrix of Spearman correlation coefficients and corresponding *p*-values for all tested pairs of differential genera and metabolites is available in Supplementary Table S9.

## 4 Discussion

**Figure 9:**
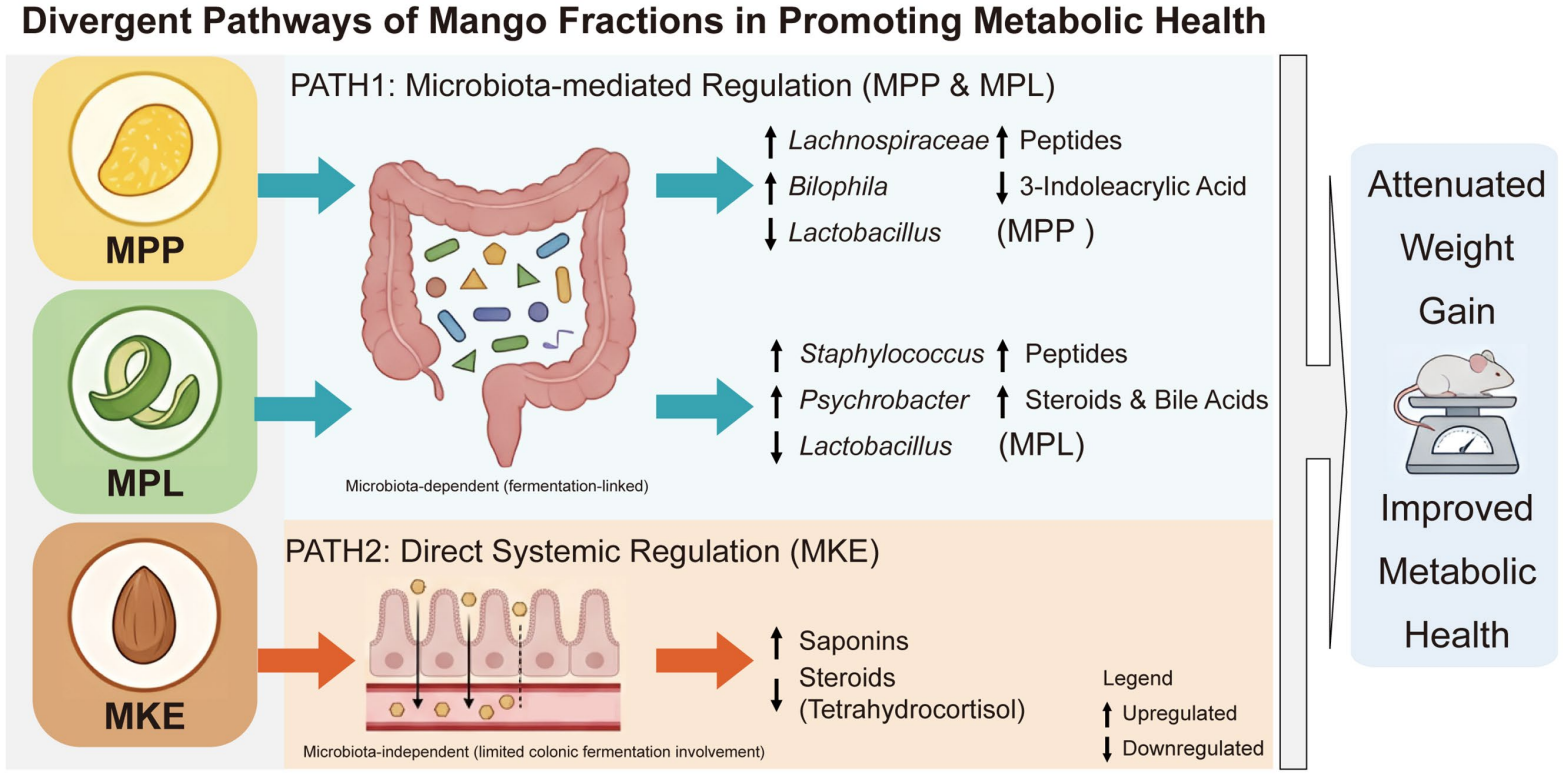
The dual-mechanism model of mango fractions in promoting metabolic health. Path 1 (microbiota-mediated regulation): MPP and MPL act by remodeling the gut microbiota. MPP enriches taxa like *Lachnospiraceae*, altering host peptide levels. MPL potently enriches *Staphylococcus* and *Psychrobacter*, which correlates with broad changes in host peptide and steroid metabolism. Path 2 (direct systemic regulation): MKE functions via a microbiota-independent (limited colonic fermentation involvement) pathway. Its bioactive saponins are absorbed to directly inhibit host steroid biosynthesis, as evidenced by decreased tetrahydrocortisol. These divergent pathways converge to attenuate weight gain and improve metabolic health. Arrows indicate upregulation (↑) or downregulation (↓).

This study integrated multi-omics data—chemical composition, animal physiology, 16S rRNA gene sequencing, and untargeted metabolomics—to dissect the distinct mechanisms by which MPP, MPL, and MKE regulate body weight. Our central finding is the confirmation of a dual-mechanism model (Figure 9): the MPP and MPL operate via a microbiota-mediated pathway, while the MKE functions through a direct, systemic pathway involving the absorption of its bioactive compounds.

### 4.1 MPP: modulates host metabolism by selectively nourishing gut bacteria

The mechanism of MPP aligns with the established pathway for fruits rich in dietary fibers and polyphenols (9, 11). MPP intervention increased gut microbiota *α*-diversity, a common indicator of a healthy microecology (Figure 2) (18), and selectively promoted the growth of bacteria associated with fiber fermentation and bile acid metabolism, such as the family Lachnospiraceae (19) and the genus *Bilophila* (Figure 3) (20).

This targeted microbial enrichment drove functional metabolic changes in the host. The correlation analysis provided a direct link: the MPP-enriched Ruminococcaceae family (the higher-level taxon for Lachnospiraceae) was strongly and positively correlated with specific lipid-like molecules (Figure 8). This suggests that the MPP’s components serve as preferred substrates for these bacteria, whose subsequent metabolic activities alter the host’s lipid profile. This alteration is likely an upstream event that contributes to broader metabolic shifts, such as the observed changes in peptide metabolism (Figure 6). Thus, our findings suggest that MPP’s moderate weight-Ctrl effect is associated with the selective enrichment of a specific subset of gut microbiota, potentially by serving as a nutrient source for them (Figure 9, Path 1).

### 4.2 MPL: drives potent metabolic changes by inducing a bloom of specific bacteria

The MPL induced a more profound and extensive alteration of the gut microecology, demonstrating a significantly more potent version of the microbiota-mediated mechanism.

A key feature of the MPL intervention was its unique ability to induce a “bloom” of specific low-abundance taxa, elevating genera like Staphylococcus and Psychrobacter to prominence (Figure 3). While typically known as skin commensals (21, 22), the bloom of Staphylococcus and its correlation with tripeptides suggests a novel metabolic role in the gut. Even more striking was the bloom of *Psychrobacter*, a genus typically found in cold environments (23, 24). Our correlation analysis provided the “smoking gun”: the bloom of Staphylococcus was positively correlated with upregulated host tripeptides, while the increase in *Psychrobacter* was strongly correlated with a wide array of steroid and bile acid metabolites (Figure 8). This directly explains the MPL’s powerful and widespread influence on host lipid and peptide metabolism (Figure 6). Therefore, we hypothesize that the MPL’s superior weight-control effect is linked to its capacity to cultivate specific bacteria, which in turn are associated with a vigorous re-engineering of the host’s metabolic landscape (Figure 9, Path 1).

### 4.3 The ecological context of *Lactobacillus* suppression

A particularly noteworthy finding from our study is the significant suppression of the genus *Lactobacillus* by both MPP and MPL interventions, which may seem counterintuitive given that lactobacilli are often considered beneficial. However, this result should be interpreted within the broader ecological context of the gut. One plausible biological consequence is simply ecological competition. More importantly, this shift may represent a functional maturation of the gut ecosystem. *Lactobacillus* species are often considered ‘generalist’ and rapid colonizers, thriving on simpler substrates. The introduction of complex polysaccharides from MPP and MPL likely created a selective pressure favoring ‘specialist’ fermenters (such as Lachnospiraceae and the taxa enriched by MPL), which are better equipped to utilize these specific nutrients. Therefore, the reduction in *Lactobacillus*, when coupled with the observed increase in overall microbial diversity and the production of beneficial metabolites like peptides, does not necessarily indicate a harmful suppression. Instead, it likely reflects a transition from a simpler community to a more complex and functionally specialized ecosystem, which ultimately contributed to the beneficial physiological outcomes.

Furthermore, as the reviewer astutely points out, this effect could be highly strain-specific, and our interpretation is constrained by the resolution of our methodology. The genus *Lactobacillus* is functionally diverse, containing numerous species and strains with varied metabolic capabilities and host effects. The V3-V4 region of the 16S rRNA gene, while effective for genus-level identification, lacks the resolution to distinguish between these closely related species or strains. Therefore, we cannot discern whether the observed suppression targeted a specific, perhaps less beneficial or overly dominant species within the healthy mouse gut, or the entire genus. Ultimately, the net physiological outcomes—including increased overall microbial diversity and attenuated weight gain—suggest that the community-wide remodeling induced by MPP and MPL was beneficial to the host, despite the reduction in the relative abundance of this particular genus. Clarifying the species- and strain-level dynamics of this suppression remains a key objective for future research using shotgun metagenomics.

### 4.4 MKE: a systemic regulator acting via a largely microbiota-independent mechanism

The most striking finding of this study is the distinct mechanism of MKE, which appears to be largely independent of colonic microbial modulation. The apparent paradox—that MKE was the most effective component at inhibiting weight gain yet induced minimal changes in the fecal metabolome—is resolved by the “direct action” hypothesis (Figure 9, Path 2), which is supported by a confluence of evidence. We established a direct molecular chain of evidence linking an exogenous plant compound to a specific host endocrine pathway: elevated levels of MKE-derived bioactive compounds, such as saponins, were detected in the host, coinciding with a significant decrease in an endogenous host steroid metabolite, Tetrahydrocortisol. This aligns with previous studies demonstrating that certain saponins can be absorbed and interfere with steroidogenic pathways (25, 26). This hypothesis is powerfully substantiated by a decisive lack of microbe-metabolite correlation. Our analysis revealed no significant statistical link between the MKE’s primary microbial impact (the suppression of Muribaculaceae, a family of known polysaccharide degraders) (27, 28) and its primary metabolic outcome (the suppression of host steroid pathways). This statistical disconnect is a critical finding, as it is consistent with the hypothesis that the microbial shifts are not the primary drivers of the MKE’s main physiological effect. Therefore, we propose that MKE’s primary mechanism is likely systemic metabolic regulation, where its bioactive compounds are absorbed by the host to directly inhibit steroid synthesis pathways (25, 26), while its impact on the microbiota represents a secondary effect rather than the cause of its potent weight-inhibiting benefits.

### 4.5 Limitations and future perspectives

While this study provides strong multi-omics evidence for a dual-mechanism model, several limitations must be acknowledged to contextualize the findings appropriately.

First, a notable limitation is the reduced sample size used for the omics analyses (*n =* 4 for microbiota, *n =* 6 for metabolomics) from the larger experimental cohort (*n =* 12). This pragmatic decision, driven by budgetary constraints, has important implications for statistical power. While we attempted to mitigate selection bias by choosing samples most representative of the group’s average physiological response (weight gain), the small sample size, particularly for the microbiome analysis, inherently limits the ability to detect more subtle biological effects and may not fully capture the spectrum of inter-individual variability. Therefore, our findings should be interpreted as highlighting the most dominant and robust effects of the mango interventions. Future studies with larger sample sizes are essential to validate these results and to uncover less pronounced, yet potentially significant, microbial and metabolic shifts.

Second, and most importantly, the conclusions drawn from this study are fundamentally associative, not causal. As correctly highlighted by the reviewer, 16S rRNA-based correlation analysis, while powerful for generating hypotheses, cannot establish a definitive cause-and-effect relationship. For example, while we observed that MPL administration was associated with a bloom of Staphylococcus and changes in peptide metabolism, we cannot conclude from this data alone that the bacteria caused these metabolic shifts.

Consequently, establishing causality for the proposed mechanisms is a critical next step. This would require dedicated experimental approaches, as suggested by the reviewer. Future studies should include interventions in germ-free mice to confirm the microbiota-dependent effects of MPP and MPL, fecal microbiota transplantation (FMT) from treated donors to naive recipients to test the sufficiency of the altered microbiota, and experiments with isolated bioactive compounds (such as the saponins from MKE) to validate their direct systemic effects. Such studies are essential to move from the hypothesis-generating phase of our current work to mechanistic validation.

Finally, the study has other methodological limitations that open avenues for future research. As a mouse model study, these findings require eventual validation in human clinical trials to confirm their relevance. The use of 16S rRNA sequencing offers limited taxonomic and functional resolution compared to shotgun metagenomics, which could provide more detailed insights into the specific species and genes involved (29). Additionally, while our extraction protocol for MKE is standardized, the final extract was not quantitatively characterized for its key bioactive compounds (e.g., total saponin content). Future studies should include this quantification to enhance reproducibility and more precisely link specific dosages of bioactives to physiological outcomes. Furthermore, our metabolomics analysis was confined to fecal samples. Incorporating serum metabolomics would be invaluable for directly testing the hypothesis of systemic absorption and characterizing the pharmacokinetics of MKE components (30, 31).

## 5 Conclusion

This study reveals that mango components regulate weight via two distinct mechanisms, as illustrated in Figure 9. The MPP and MPL act on the gut microbiota, enriching for specific bacteria that alter host peptide and lipid metabolism, with the MPL being more potent. In contrast, the MKE functions through a direct, systemic mechanism. Its bioactive saponins are absorbed by the host to inhibit the endogenous steroid hormone biosynthesis pathway, an effect largely independent of major colonic microbial shifts. These findings provide a scientific basis for developing mango by-products, particularly the MPL and MKE, into high-value functional ingredients for metabolic health.

## Data Availability

The data presented in this study are publicly available. The data can be found here: https://www.ncbi.nlm.nih.gov, accession PRJNA1435576.

## Footnotes

1
www.majorbio.com

## Supplementary material

Supplementary Figure S1 Beta diversity analysis of the gut microbiota based on UniFrac distances. This figure illustrates the structural differences in the gut microbial communities among the different treatment groups. **(A)** Principal Coordinate Analysis (PCoA) based on the weighted UniFrac distance, which emphasizes changes in the abundance of dominant taxa. The analysis shows no significant separation between the groups (PERMANOVA, R² = 0.2747, *p* = 0.097), suggesting that the interventions did not significantly alter the structure of the most abundant phylogenetic lineages. **(B)** PCoA based on the unweighted UniFrac distance, which is more sensitive to the presence or absence of rare taxa. In contrast, this analysis reveals a statistically significant separation among the groups (PERMANOVA, R² = 0.2925, *p* = 0.015), indicating that the mango components induced changes in community composition, particularly affecting the presence or absence of specific, including less abundant, phylogenetic lineages. Each point represents an individual mouse sample, and the ellipses represent the 95% confidence interval for each group.
