# Supplementary figures and images for: Divergent pathways of mango fractions in promoting metabolic health: from gut microbiota remodeling to direct systemic regulation
Source: Front Nutr. 2026 Mar 23;13:1744331. doi: 10.3389/fnut.2026.1744331 (PMC13050896; doi:10.3389/fnut.2026.1744331)

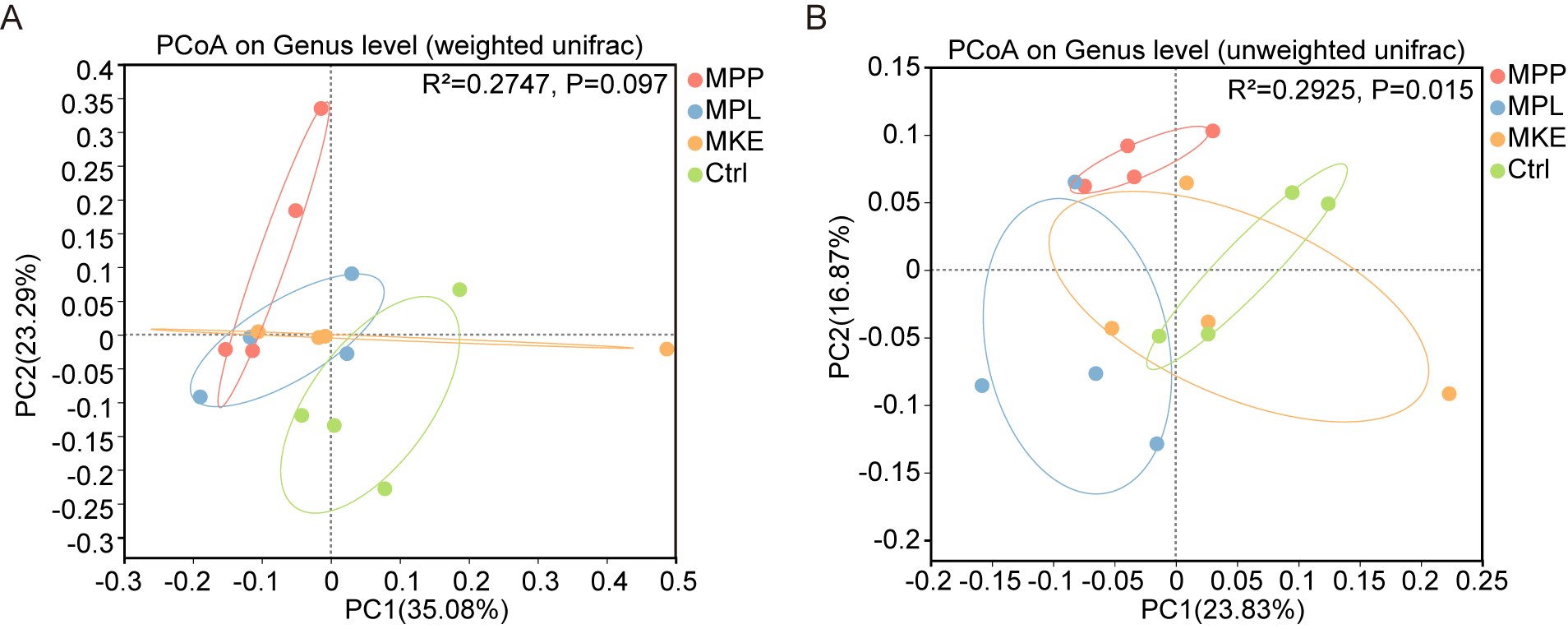

Supplement: Supplementary Figure S1 — Beta diversity analysis of the gut microbiota based on UniFrac distances. This figure illustrates the structural differences in the gut microbial communities among the different treatment groups. (A) Principal Coordinate Analysis (PCoA) based on the weighted UniFrac distance, which emphasizes changes in the abundance of dominant taxa. The analysis shows no significant separation between the groups (PERMANOVA, R² = 0.2747, p = 0.097), suggesting that the interventions did not significantly alter the structure of the most abundant phylogenetic lineages. (B) PCoA based on the unweighted UniFrac distance, which is more sensitive to the presence or absence of rare taxa. In contrast, this analysis reveals a statistically significant separation among the groups (PERMANOVA, R² = 0.2925, p = 0.015), indicating that the mango components induced changes in community composition, particularly affecting the presence or absence of specific, including less abundant, phylogenetic lineages. Each point represents an individual mouse sample, and the ellipses represent the 95% confidence interval for each group. [file Image_1.tif]
